# Pharmacovigilance analysis of the Vigibase on antidepressants-related withdrawal syndrome in adults and adolescents

**DOI:** 10.1192/j.eurpsy.2023.277

**Published:** 2023-07-19

**Authors:** C. Gastaldon, G. Schoretsanitis, E. Arzenton, E. Raschi, D. Papola, G. Ostuzzi, U. Moretti, E. Seifritz, J. M. Kane, G. Trifirò, C. Barbui

**Affiliations:** 1 WHO Collaborating Centre for Research and Training in Mental Health and Service Evaluation Department of Neuroscience, Biomedicine and Movement Sciences Section of Psychiatry, University of Verona, Verona, Italy; 2 Department of Psychiatry, Northwell Health, The Zucker Hillside Hospital, Glen Oaks, New York, USA, United States; 3 Section of Pharmacology, Department of Diagnostics and Public Health, University of Verona, Verona; 4 Pharmacology Unit, Department of Medical and Surgical Sciences, University of Bologna, Bologna, Italy; 5 Department of Psychiatry, Psychotherapy and Psychosomatics, Hospital of Psychiatry, University of Zurich, Zurich, Switzerland; 6 Department of Psychiatry, Northwell Health, The Zucker Hillside Hospital, Glen Oaks, New York, USA, United States

## Abstract

**Introduction:**

Antidepressant discontinuation may cause withdrawal syndrome in some cases. However, evidence on this syndrome related to individual antidepressants is limited, as well as about individual risk factors for severe reactions.

**Objectives:**

To ascertain whether each individual antidepressant is associated with an increased reporting of withdrawal syndrome as compared with other medications, and to examine clinical risk factors for severe reactions.

**Methods:**

We conducted a pharmacovigilance study, with a case/non-case design. We included reports of antidepressant-related withdrawal syndrome from the VigiBase, the WHO global database of individual case safety reports of suspected adverse drug reactions. We performed a disproportionality analysis (calculating reporting odds ratio (ROR) and the Bayesian information component (IC)) of reports of antidepressant-related withdrawal syndrome, comparing antidepressants to all other drugs and to buprenorphine (as a positive control). Antidepressants with significant disproportionate reporting were ranked in terms of clinical priority. We compared serious versus non-serious reactions to determine clinical risk factors for severe reactions.

**Results:**

Based on 31,688 reports of antidepressant-related withdrawal syndrome, we detected a disproportionate reporting for 23 antidepressants. The ROR for antidepressants altogether, compared to all other drugs, was 14.26 (95%CI:14.08-14.45), 17.01 for other antidepressants (95%CI:16.73-17.29), 13.65 for SSRIs (95%CI:13.41-13.90) and 2.8 for tricyclics (95%CI:2.59-3.02). Based on clinical priority ranking, the strongest signals were found for paroxetine, duloxetine, venlafaxine and desvenlafaxine (figure 1), being comparable to buprenorphine. Severe reactions were more frequently reported in males, adolescents, persons with multiple medications, and with longer treatment duration.

**Image:**

**Figure:**
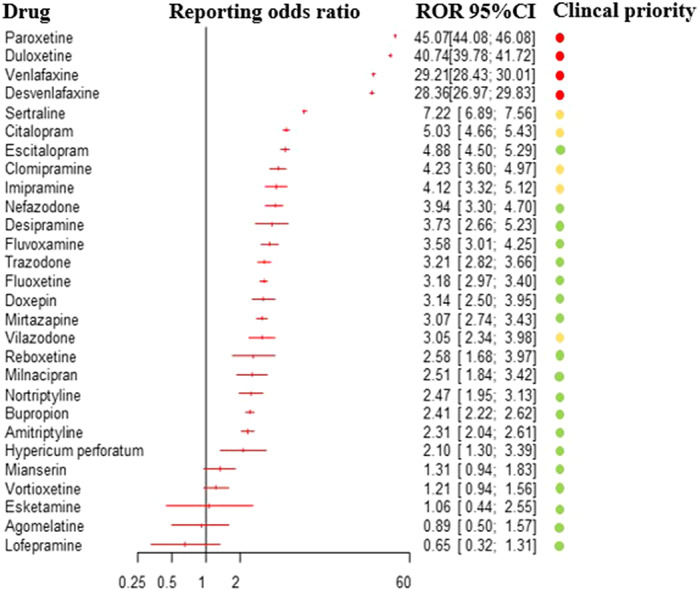

**Conclusions:**

Antidepressants are associated with increased reporting of withdrawal syndrome compared with other medications, with differences between individual antidepressants. Clinicians should be aware of such differences, when prescribing and discontinuing these drugs, as well as of the risk to experience more severe withdrawal symptoms in specific cases.

**Disclosure of Interest:**

C. Gastaldon: None Declared, G. Schoretsanitis Consultant of: Dr. Schoretsanitis has served as a consultant for HLS Therapeutics, E. Arzenton : None Declared, E. Raschi : None Declared, D. Papola: None Declared, G. Ostuzzi: None Declared, U. Moretti: None Declared, E. Seifritz Grant / Research support from: Dr. Seifritz has received educational grants, consulting fees and lecture honoraria from Janssen Cilag, Lundbeck, Angelini, Otsuka, Servier, Ricordati, Vifor, Sunovion, Schwabe and Mepha, Consultant of: Dr. Seifritz has received educational grants, consulting fees and lecture honoraria from Janssen Cilag, Lundbeck, Angelini, Otsuka, Servier, Ricordati, Vifor, Sunovion, Schwabe and Mepha, J. Kane Shareolder of: LB Pharmaceuticals and Vanguard Research Group, Consultant of: Dr. Kane has been a consultant and/or advisor for or has received honoraria from Alkermes, Allergan, LB Pharmaceuticals, H. Lundbeck, Intracellular Therapies, Janssen Pharmaceuticals, Johnson and Johnson, Merck, Minerva, Neurocrine, Newron, Otsuka, Pierre Fabre, Reviva, Roche, Sumitomo Dainippon, Sunovion, Takeda, Teva and UpToDate, G. Trifirò Grant / Research support from: he was the scientific director of a Master program on pharmacovigilance, pharmacoepidemiology and real-world evidence which has received non-conditional grant from various pharmaceutical companies; he coordinated a pharmacoepidemiology team at the University of Messina until Oct 2020, which has received funding for conducting observational studies from various pharmaceutical companies (Boehringer Ingelheim, Daichii Sankyo, PTC Pharmaceuticals). He is also scientific coordinator of the academic spin-off “INSPIRE srl” which has received funding for conducting observational studies from contract research organizations (RTI Health Solutions, Pharmo Institute N.V.)., Consultant of: Dr. Trifirò has served in the last three years on advisory boards/seminars funded by SANOFI, Eli Lilly, AstraZeneca, Abbvie, Servier, Mylan, Gilead, Amgen;, Speakers bureau of: Dr. Trifirò has served in the last three years on advisory boards/seminars funded by SANOFI, Eli Lilly, AstraZeneca, Abbvie, Servier, Mylan, Gilead, Amgen;, C. Barbui: None Declared

